# Ethereal Tinct

**Published:** 1860-09

**Authors:** B. W. Hardee

**Affiliations:** Savannah, Ga.


### ARTICLE IV.

### *Ethereal Tinct. Valerian in Convulsions in Children.* By B. W. HARDEE, M. D., Savannah, Ga.

Physicians are recognised as a class of men whose duty it is to relieve suffering humanity of its diseases, as well as to find out means for the permanent cure and prevention of diseases.

Now, in writing this communication, I do not claim or pretend to say that I have made a discovery in the use of Ethereal Tinct. Valarian in convulsions among children, as an invaluable remedial agent, but my desire is merely to call the attention of the profession to the use of this medicine in convulsion. From the fact of its having been so beneficial in several cases in which I have used it, I am induced to think that it has few, if any, equals in arresting convulsions. I have employed it as an anti-spasmodic in several cases, with entire satisfaction to myself and relief to my patient. There may be a doubt as to the propriety of using the Ethereal Tinct. Valerian in cases of convulsions in young children. The cases which have fallen under my care have all been in children from one to three years old.

Convulsions among children is not a very fatal disease, unless complicated with some functional disorder. The causes of convulsions are very numerous. The state of the nervous system in childhood is said to be a predisposing cause; a weak and anemic state of the systems predisposes to convulsions; fear, anger, surprise, or any very sudden emotion, exposure to heat or cold, over-exertion, falls, &c., are sometimes the causes of convulsions; but irritation transmitted to the brain, either from teething, or from intestinal worms, or indigestible food—in fact, whatever occasions spasm in the intestines, may induce convulsions. The predispositions in some children are so great that they never have the slightest attack of fever without having several convulsions.

I shall now proceed to give an account of some cases which fell under my observation for treatment:

October 13, 1858, I was called to see a little child of Mr. S., a few miles from town. Upon my arrival I found a little girl, age about three years, in convulsions. Her mother, quite an intelligent lady, informed me that she had had two convulsions; that they had lasted but about five minutes. Her pulse was frequent and quick, her bowels constipated, her tongue was coated with a white fur, and a little red upon the edges. Upon examining her very carefully, and upon asking her mother several questions, I decided that the convulsions were probably brought about by worms in the intestinal canal, causing irritation there. I immediately ordered that she should take six drops of the Ethereal Tinct. Valerian every fifteen minutes, until all twitching of the mus cles should cease. I also gave an enama of Flaxseed Tea, and about forty drops of Valerian in it. I remained to watch effects, and as soon as all twitching of the muscles had ceased, ordered the following powders to be given every hour:

℞—Hydrarg. chlor. mit. grs. iij.
Pulv. spigelic, grs. viij.
M. ft. chart No. iij.

To be followed in two hours after last powder is taken by a dose of castor oil and turpentine.

On the 14th I called to see my little patient, and found her improved. She had one convulsion during the night. The medicine had operated, and a number of worms passed from her; but still there was a disposition to convulsion. I

then ordered that she should take four drops of Ethereal Tinct. Valerian every hour. She also had had some fever that night. I ordered Quinine in one grain doses to be given every hour until she had taken five doses.

On the 15th I visited her again. She was much improved; all symptoms of convulsions had subsided, and I discharged her.

*Case 2d.*—April 16th, 1858, I was summoned by Mrs. P. to see a little negro boy, belonging to R. S.—age, four years. Upon my arrival, I found the boy in a severe convulsion; he had been in that condition two hours. He had shown no symptoms of being sick, but was taken down with the convulsion very suddenly. I was unable to find out the state of his bowels. His stomach was hard and swollen; his pulse numbered one hundred and forty—quick and strong. I ordered an enama of warm water and Valerian—a teaspoonful of Valerian to four ounces of water—and administered it, and gave him six drops by the mouth. As soon as the enama acted upon him he recovered, but appeared very weak. I ordered a little brandy toddy, and also four drops of Valerian, every half hour, until all motion of muscle ceased, and then gave him the following powders:

℞—Pulv. spigelii, grs. xx.

Make four powders, and give one every hour. He had several actions from his bowels with a few worms.

On the 17th I called again. He had not had any more convulsions, his skin soft, his bowels open, his pulse about one hundred and ten, very weak. I then ordered the following powders:

℞—Ferro Cyancite Quinia, x. grs.
M. ft. Pulv. Doveri, grs. ij.

Four powders, one every two hours.

On the 18th I called again. He was much improved, suffering from weakness. I ordered a continuation of powders, with a little toddy occasionally; and on the 19th I called again and discharged him.

I have had several cases of a similar character, but not knowing at the time I was treating them that I would publish them, I did not take any notes; but in nearly all cases I have had, I have used the Ethereal Tinct. of Valerian with the very best results.

